# Referral challenges for early-onset colorectal cancer: a qualitative study in UK primary care

**DOI:** 10.3399/BJGPO.2023.0123

**Published:** 2023-10-18

**Authors:** Orla M O'Neill, Helen G Coleman, Helen Reid

**Affiliations:** 1 Cancer Epidemiology Research Group, Centre for Public Health, Queen’s University Belfast, Belfast, Northern Ireland, UK; 2 Dunluce Health Centre, Belfast, Northern Ireland, UK; 3 Patrick G Johnston Centre for Cancer Research, Queen’s University Belfast, Belfast, Northern Ireland, UK; 4 Centre for Medical Education, Queen’s University Belfast, Belfast, Northern Ireland, UK

**Keywords:** colorectal neoplasms, delayed diagnosis, general practice, incidence, Northern Ireland, primary health care, qFIT, qualitative research, referral and consultation

## Abstract

**Background:**

The incidence of early-onset colorectal cancer (EOCRC) in adults aged <50 years has increased in several Western nations. National surveys have highlighted significant barriers to accessing timely care for patients with EOCRC, which may be contributing to a late stage of presentation in this population group.

**Aim:**

To explore awareness of the increasing incidence of EOCRC, and to understand the potential barriers or facilitators faced by GPs when referring younger adults to secondary care with features indicative of EOCRC.

**Design & setting:**

Qualitative methodology, via virtual semi-structured interviews with 17 GPs in Northern Ireland.

**Method:**

Reflective thematic analysis was conducted with reference to Braun and Clarke’s framework.

**Results:**

Three main themes were identified among participating GPs: awareness, diagnostic, and referral challenges. Awareness challenges focused on perceptions of EOCRC being solely associated with hereditary cancer syndromes, and colorectal cancer being a condition of older adults. Key diagnostic challenges centred around the commonality of lower gastrointestinal complaints and overlap in EOCRC symptoms with benign conditions. Restrictions in age-based referral guidance and a GP ‘guilt complex’ surrounding over-referral to secondary care summarised the referral challenges. Young females were perceived as being particularly disadvantaged with regard to delays in diagnosis.

**Conclusion:**

This novel research outlines potential reasons for the diagnostic delays seen in patients with EOCRC from a GP perspective, and highlights many of the complicating factors that contribute to the diagnostic process.

## How this fits in

The incidence of early-onset colorectal cancer (EOCRC) has risen in many Western countries in recent years. Patients with EOCRC have been shown to experience longer diagnostic delays than their older counterparts. This research highlights the perceived awareness, diagnostic, and referral challenges faced by GPs when younger adults present to primary care with lower gastrointestinal tract symptoms indicative of EOCRC.

## Introduction

The incidence of EOCRC, defined as affecting adults aged <50 years, has increased substantially in recent decades in many Western countries.^1,2^ A 2022 rapid review demonstrated consistent evidence of the rising trends seen globally, highlighting the need for change to suspected cancer referral guidance.^3^ In the UK, incidence rates of EOCRC in adults aged 25–49 years increased by 48% from 1993–2018.^4^ Furthermore, global mortality rates from EOCRC increased by 18% from 1990–2019.^5^ By 2030, it is projected that EOCRC will be the leading cause of cancer-related deaths in individuals aged 20–49 years in the US.^6^

A body of research has indicated a birth cohort effect in relation to people born after the 1960s having an increased risk of EOCRC.^7^ Although multiple factors implicated in EOCRC have been widely debated, such as the increasing prevalence of obesity, low levels of physical activity, high processed food intake, or altered gut microbiota, there have been few conclusive findings.^8–10^ The vast majority of EOCRC diagnoses are not associated with an inherited cancer syndrome, and therefore sporadic, symptomatic presentation is the most common route for diagnosis.^11,12^

Abdominal pain has been found to be a pertinent presenting feature in patients with EOCRC, when accompanied by rectal bleeding and a change of bowel habit.^13–15^ For this reason, the 2015 National Institute for Health and Care Excellence (NICE) guidance included criteria for individuals aged <50 years presenting with rectal bleeding, to include associated abdominal pain as a possible red flag cancer symptom.^16^

A survey carried out by Bowel Cancer UK as part of their Never Too Young campaign demonstrated that four in ten patients who had asked their GP if their symptoms could be caused by bowel cancer were told that they were ‘too young’ for that to be the case.^17^ A population-based study in England highlighted the referral challenges faced by patients with EOCRC, with only 22.6% being referred through a 2-week wait (red flag) referral pathway. The study theorised that the reduced suspicion of cancer among healthcare professionals, as well as limitations in age-based referral guidelines, resulted in young patients being referred through a non-urgent pathway.^13^

With the high volume of consultations within primary care, the challenge for GPs is distinguishing the rarer EOCRC cases for referral. Lower gastrointestinal tract symptoms account for one in twelve consultations in primary care, reflecting the common nature of gastrointestinal complaints.^18^ The overlap in symptomatology when patients present with undifferentiated symptoms shared with benign illness further contributes to the complexity of diagnosing cancer in primary care.^19^

This research aimed to explore awareness of the increasing incidence of EOCRC, and to understand the potential barriers or facilitators faced by GPs when referring younger adults to secondary care with features indicative of EOCRC.

## Method

Virtual semi-structured interviews were conducted with 17 GPs in Northern Ireland. Ethical approval was obtained from the Faculty of Medicine, Health, and Life Sciences Research Ethics Committee at Queen’s University Belfast (reference number: MHLS 21_55).

### Participant selection and characteristics

Participants were limited to GPs practising within Northern Ireland, owing to specific regional referral guidance being used throughout the UK. Invitations (see Supplementary Information S1) to participate in semi-structured interviews were disseminated widely via Northern Ireland GP mailing lists. This included practice manager and out-of-hours coordinator networks, the Royal College of General Practitioners Northern Ireland newsletter, and a closed Northern Ireland primary care social media group, as approved by the ethics committee. Participation was voluntary and not incentivised. Twenty-three participants actively contacted the research team to express interest in participating in the study. Interviews were scheduled at participant convenience once the required written consent forms were completed and submitted. With transcription and preliminary analysis happening in parallel with further interviews, it was possible to make rational decisions around data sufficiency. This point, with paucity of novelty, was achieved after 17 interviews.

### Data collection

The interview guide was collaboratively developed by the research team, using their combined expertise in qualitative research, epidemiology, and general practice, alongside patient and public involvement from the Northern Ireland Cancer Research Consumer Forum. The final interview guide was piloted with GP academic colleagues at Queen’s University Belfast, with the clarity of the study’s aim to include facilitators as well as barriers to referral addressed following piloting. The interview guide included six broad questions (see Supplementary Information S2). Although the interview guide provided a framework, discussions were typically guided by participant responses.

Virtual semi-structured interviews were conducted by the primary researcher (OO’N) and recorded over Microsoft Teams between June and July 2021. Interviews lasted between 20 and 43 minutes, with a median time of 31 minutes.

Interviews took place during a time of change to Northern Ireland Cancer Network (NICaN) referral guidelines in June 2021.^20^ Two key updates to NICaN guidelines at this time were the removal of age restrictions for referrals, and the implementation of quantitative faecal immunochemical test (qFIT)-based triage.^21^

### Data analysis

Data were analysed using the framework of Braun and Clarke’s thematic analysis.^22^ Interviews were transcribed verbatim by OO’N. Transcription notation was adapted from formal conversational analysis transcribing conventions.^23^ Further data familiarisation was achieved through repeated readings of the transcripts by OO’N and preliminary coding on a case-by-case basis. Four transcripts were picked at random and analysed independently by HR to compare and discuss codes in a reflective and collaborative manner. This enabled richer interpretations and provided a consensus on the application of codes to subsequent transcripts. OO’N then coded the data on a cross-case analysis basis, and grouped codes inductively into subthemes. The research team analysed the subthemes and together actively identified themes in an inductive process, identifying those most relevant to the research question.

## Results

Demographics of the 17 participating GPs are shown in Table 1. Participating GPs represented a relatively even mix of sexes, ages, and practice demographics (rural, urban, or mixed). The majority of participating GPs had been qualified for ≥11 years (Table 1).

**Table 1.** Demographics of GPs participating in interviews

Characteristic	*n* (%)
Sex	
Female	9 (53)
Male	8 (47)
Age, years	
30–40	5 (29)
41–50	5 (29)
51–60	5 (29)
>60	2 (12)
Years qualified	
0–10	1 (6)
11–20	7 (41)
21–30	7 (41)
>30	2 (12)
Practice demographic	
Rural	4 (24)
Urban	6 (35)
Mixed	7 (41)

Three main themes were identified from the data on awareness, diagnostic, and referral challenges. Awareness subthemes included the perception of colorectal cancer (CRC) as a disease of older adults, as well as a presumption that it is a hereditary cancer syndrome in younger adults. Diagnostic challenges reflected the common nature of lower gastrointestinal tract complaints presenting to general practice, and overlap with benign pathologies. A theme was also identified of young females being disadvantaged with regard to delays in diagnosis. Referral challenges included age-based guideline constraints and a ‘guilt complex’ felt by GPs when referring patients to a poorly resourced secondary care system.

### Awareness challenges: never too young

#### Disease of older adults

CRC was perceived by the majority of the participants to be a disease of older adults, providing insight into why younger patients may be more likely to experience delays in referral for further investigation:

*‘… I suppose my radar would be mainly in the older population.’* (Participant [P]13)

*‘I still think for most GPs age is a great reassurer … particularly in the 20s and 30s, I mean sometimes the late 40s … people think emm benign, or inflammatory bowel disease, you don’t think about cancer*.*’* (P2)

With participants less likely to consider the diagnosis in their immediate differentials of a younger patient, they highlighted the diagnostic uncertainty based on symptoms alone:

*‘I am less likely to search for it in the much younger population … there’s also the risk of overlap with the inflammatory bowel disease as some of the symptoms can be similar*.*’* (P9)

Perceptions were influenced by participants’ pre-existing knowledge of CRC, as well as local, age-based referral guidelines:

*‘I think eh … less than 40 and really emm … you could go up as far as 50 but I would say definitely less than 40 I really need, I need to be convinced, that I need to refer them you know … My threshold is higher certainly*.*’* (P1)

*‘Yeah obviously the over 50s it’s easier again because it’s so much more easily quotable from the guidelines.’* (P14)

#### Presumed hereditary cancer syndrome

There was a general perception that EOCRC was associated with hereditary or genetic cancer syndromes, and therefore patients without a significant family history were less readily referred:

*‘… he had the Lynch syndrome, that would be sort of … in my head thinking of somebody who’s very young, be related to a genetic condition.’* (P17)

*‘If there was a significant family history then they would be most likely getting referred on as well ... you know maybe wouldn’t be hanging about as much to see if things resolve ... you know I’d probably refer earlier if there was that kind of thing.’* (P1)

One participant reflected on the routine referral of an EOCRC patient with rectal bleeding and a family history of bowel cancer, rather than not referring at all, owing to a perceived benign diagnosis in a young patient:

*‘… the difficulty was I referred him routinely rather than red flag, and one of the reasons was his mother had bowel cancer and I felt we were dealing with something like haemorrhoids here, so I did routine referral probably more on the basis of his mother had bowel cancer, now it wasn’t a particularly bad family history it just seemed to be almost coincidental*.*’* (P15)

### Diagnostic challenges: the great imitator

#### Commonality of gastrointestinal complaints

The complexity of identifying patients with EOCRC in general practice focused on the large numbers of presentations of lower gastrointestinal tract complaints with benign aetiology, making it difficult to identify those with sinister diagnoses:

*‘I guess that’s the problem, you're absolutely swamped with presentations.’* (P10)

*‘… a very very common presentation and that can make it quite difficult to you know … you have your differentials … and make a decision as to what is the most likely.’* (P17)

#### Overlap with benign pathologies

Participants reflected on the vague presentation and typical overlap of symptoms with more common benign conditions in this age group, such as irritable bowel syndrome (IBS), inflammatory bowel disease, and benign anorectal conditions such as haemorrhoids. Participants highlighted the difficulty of differentiating these conditions from malignant disease with the investigations and tools available in primary care:

*‘You get so many of the other sort of irritable bowel type patients with any sort of quite mild symptoms and they’re in contact quite frequently you know so it’s … eh I don’t know, it’s sort of that deluge of patients and sort of trying to work out which are the patients that … that have concern.’* (P10)

*‘It’s in that group, the diagnostic uncertainty, the chances are it’s going to be something else, emm and being able to tease out, with I suppose the tools that we have.’* (P12)

*‘My impression is that in younger people it seems to present later, maybe … I don’t know if that’s because it tends to be more aggressive or it’s just that the sort of, you know, your suspicion isn’t as raised and maybe you’re more quick to say people have IBS or whatever.’* (P11)

#### Females most disadvantaged

Several GPs independently acknowledged that young females were most disadvantaged when it came to delays in referral and diagnosis. Potential reasons for this sex disparity were discussed by participants:

*‘I think the under 50s emm … women with iron deficiency anaemia are often, eh sort of signposted or … put into the pigeonhole of menstrual losses ... the altered bowel habit as well isn’t necessarily as big a red flag, and ultimately it usually takes longer before you refer simply because of the overlap with irritable bowel type things.’* (P2)

*‘Women who are under 50 who are still menstruating as well who have iron deficiency anaemia, then you’ll just put it down to that and then you’re less likely to investigate as well so you know that’s another I suppose risk factor for that being missed.’* (P11)

*‘… with females in particular emm, gynae-related issues can be … it can be difficult to discern between gynae problems and what can be bowel-related problems*.*’* (P17)

### Referral challenges: GP guilt and guidelines

#### GP guilt complex

Participants described a sense of guilt associated with referring patients to secondary care for further investigations, and the pressure that waiting lists had on their decision making with regard to referral:

*‘The art is trying to sift out those guys without, without over-referring, and I suppose ultimately I have, feel the … on a personal level, feel the burden of our long waiting lists and … tend to carry risk and under-refer compared to some colleagues.’* (P*2*)

*‘I think one of the problems … is that everything is getting red-flagged by you know … not from every GP, but I think probably our thresholds are too low … they must be, sometimes very exacerbated with us … it’s the system is broken … certainly in Northern Ireland, you could have all the guidelines you like, but if you don’t have the resources then what?’* (P16)

#### Guideline constraints

A significant issue highlighted by participants was the constraint that guidelines, including historic NICaN referral guidance,^20^ put on their referral-making decisions, and sometimes the downgrade of referrals if guidelines were not met:

*‘The biggest problem we have is getting these patients seen because of the referral guidelines.’* (P17)

*‘… whenever I do a referral … a red flag referral, I have to make it fit, because if I don’t it’ll come back to me … ’* (P4)

*‘… if a patient fulfils it, it makes it very straightforward but I think there’s probably quite a caveat, and especially the younger patients who don’t strictly meet the criteria and that’s the problem and you might refer them … but probably knowing that the referral may well be downgraded, which is probably our experience … ’* (P7)

In particular, age was identified as the main barrier to red flag referral, and participants reflected that younger patients often languished on non-red flag waiting lists:

*‘I think most of the guidelines are all set up to be pretty clear for the over 50s, and for the under 50s it gets increasingly less clear … There is no service for anybody who doesn’t meet red flag criteria.’* (P2)

*‘… therein lies the difficulty, no matter what your age but particularly for young people or younger patients because they often don’t tend to tick the red flag box by virtue of their age and therefore they are losing out … you know potentially we are missing significant diagnoses.’* (P12)

With the long waiting lists for secondary care within the NHS, GPs felt that patients not meeting red flag criteria were particularly disadvantaged with delays in diagnosis. Participants reflected on the impact this had on patients, as well as on GPs managing these patients in primary care:

*‘Well certainly if I’ve put in an urgent referral, I certainly hope to myself that there isn’t anything serious wrong, I’m usually aware that they don’t fit into the guidelines … but I’m aware that that doesn’t make it impossible that they have bowel cancer, and I worry about the fact that urgent referrals are taking a long time to be seen.’* (P5)

*‘The problem is you’re left with carrying the can until the diagnosis is made … at the end of the day, the patient’s my responsibility and if they were doing … you know if the red flag pathway whereby they got seen and scoped within 4–6* [weeks] *… I’m not going to be awfully worried about it but knowing what the waiting lists are like I know it’s back to me … ’* (P4)

## Discussion

### Summary

This qualitative study highlights some of the important challenges faced by GPs when assessing and referring younger patients with lower gastrointestinal tract symptoms indicative of cancer. With the worrying globally increasing trends in EOCRC, and the often late stage at diagnosis seen in these patients,^24–26^ there is a need to identify challenges and implement change to protect future generations. The interviews in this study provide an insight into factors contributing to these delays. The complexity of making a diagnosis in general practice is multifactorial. The commonality of the presentation, as well as the overlap in symptomatology with benign conditions, adds to this complexity. Furthermore, an ingrained perception of CRC being a disease of older adults, and younger patients being associated with hereditary cancer syndromes, lowers GPs’ suspicions. The barriers that primary care faces with regard to referral guidance also complicates the diagnostic process in many younger patients, compounded by long waiting lists and an underlying feeling of guilt among many GPs about over-referring to secondary care. Unique diagnostic challenges were noted, particularly around young females with potential EOCRC, such as presumptive menstrual causes for iron deficiency anaemia and females being more readily diagnosed with IBS.

### Strengths and limitations

The authors believe this to be novel research into the unique GP perspective, that builds understanding of why there might be delays in primary care with regard to patients with EOCRC in a UK region. The research design was strengthened by the mix of expertise within the research team, and robust involvement of the patient and public perspective. A methodological strength of the data analysis was the independent coding of some reports by an experienced qualitative researcher as a second reviewer.

Limitations to this research include the potential for regional specific challenges, given that the study was limited to GPs practising in Northern Ireland. However, the majority of the themes identified are likely to be theoretically transferable to other UK regions, and indeed, internationally. It is also acknowledged that the timing of the interviews occurred during a period of change to local referral guidance, with age-specific criteria being removed from red flag guidance.^21^ However, it is likely that the long-standing age-based criteria will influence decision making by GPs into the future, and ingrained perceptions could transcend guidelines.

### Comparison with existing literature

It is well documented that patients with EOCRC experience longer diagnostic delays compared with their older counterparts.^27,28^ A European retrospective case–case study found that only 40.7% of patients with EOCRC were diagnosed within 6 months from symptom onset, compared with 85.6% of patients over the age of 50 years.^29^ GPs are more likely to suspect cancer in older patients, with younger age and female sex being found to be factors that contribute to GPs’ cognitive bias with regard to their first impressions of cancer risk.^30^ Although hereditary cancer syndromes or family history of CRC are significant risk factors for the disease,^31^ the majority of EOCRC is known to be sporadic.^32^ Low levels of suspicion among GPs in young patients without a family history of CRC was identified as a potential barrier to further investigation and referral within the present study. To the authors’ knowledge, this novel finding on GPs’ misperceptions of family history and inherited cancer syndromes among patients with EOCRC has not been previously reported.

A large UK study of 36 primary care practices in the mid-1990s found that gastrointestinal symptoms accounted for one in twelve consultations, and of those symptomatic patients, nearly half were diagnosed with a functional bowel disorder including IBS.^18^ With an estimated 11% of the global population affected by IBS,^33^ the difficulty is trying to identify sinister pathology, particularly with the overlap in benign symptoms. An English data-linkage study has found that females with CRC and abdominal symptoms in the prediagnostic year were twice as likely to be diagnosed with a benign condition than males with similar symptoms.^34^ Females aged 40–59 years with a new onset benign diagnosis were at particularly high risk of an emergency presentation of their CRC diagnosis.^34^ The theme of perceptions of CRC being a diagnosis of older patients, as well as prioritisation of common conditions by GPs, were also highlighted as potential barriers to timely diagnosis of EOCRC in a UK mixed-methods study.^35^ This study also identified a possible sex disparity, with females more likely to report a misdiagnosis of IBS and psycho-emotional diagnoses such as stress.^35^

The theme of young females being disproportionately disadvantaged when it comes to delays in diagnosis has also been highlighted in other reports. Bowel Cancer UK’s Never Too Young report in 2015 found that 20% of patients with EOCRC presented to their GP ≥5 times before referral, with 54% of male patients being referred after <3 consultations, compared with only 35% of female patients.^36^ Younger females have previously been found to be more likely to experience missed opportunities for diagnosis of CRC than males^37^ and experience longer overall clinical delays.^38^ Interestingly, unlike late onset CRC, which has a slight predominance in males, rates of EOCRC have been found to be similar in both sexes.^39^ Siegel *et al* demonstrated that since 1994, both sexes have seen incidence rates increase >50%.^40^ GPs need to have increased awareness of EOCRC affecting both males and females to a similar extent. The present research has highlighted some of the potential reasons for sex differences seen with referral patterns, including iron deficiency anaemia being attributed to menstrual losses, and an overlap between bowel and gynaecological symptomatology.

Another highlighted theme identified guideline constraints and a poorly resourced secondary care system as potential barriers to onward referral. Delays within primary care relating to accessibility, referral pathways, and resources were also identified in a European cross-sectional survey as potential barriers to timely cancer diagnoses.^41^ Furthermore, the concept of a GP guilt complex with regard to referral was noted in a UK-based primary care study, which investigated all-age CRC suspicions in GPs.^42^ Among patients meeting urgent referral criteria, that study found that key barriers to referral included a fear of over-referral or unnecessary referrals, with participants wishing to avoid being identified as having a high referral rate.^42^ With NICE referral guidance having an estimated 3% cancer pick-up rate,^16^ the perception that referrals should amount to a high level of cancer diagnoses is skewed, but it is interesting that GPs feel this pressure with regard to their referrals.

Research exploring variations in suspected cancer referral pathways across ten different jurisdictions in the International Cancer Benchmarking Partnership concluded that allowing more flexibility with referral pathways, with greater access to investigations and open channels of communication with secondary care, may improve cancer diagnostic delays.^43^ The authors exemplified the Australian system as having flexibility with referrals and direct access to investigations, and as being in part responsible for their success with higher cancer survival and earlier stage at diagnosis than international counterparts.^43^ Waiting lists for secondary care within the NHS are significant at present, particularly in Northern Ireland.^44^ It is important to address accessibility to investigations and secondary care to improve the diagnostic journey for patients presenting to primary care.

### Implications for research and practice

This research aimed to gain a deeper understanding of why patients with EOCRC experience longer diagnostic delays. This research has the potential to alter practice by challenging long-standing perceptions and addressing deficits of awareness and knowledge of EOCRC among healthcare professionals. By acknowledging the unique diagnostic challenges faced by patients with EOCRC, particularly females, it is hoped that their diagnostic journey can be improved. It is imperative that GPs have access to tools to aid decision making with regard to onward referrals or improved diagnostic pathways.

The future for early diagnosis may be found in the implementation of risk stratification tools in primary care, as evaluated in a recent English cohort study incorporating blood test trends to identify patients at high risk of cancer.^45^ Future research should evaluate whether the introduction of symptomatic qFIT use in NICaN primary care referral guidance^21^ and the removal of age-based restrictions could have a potential impact on earlier diagnosis of patients with EOCRC. qFIT use in symptomatic patients has been found to have a high level of sensitivity for CRC, with the benefits of identifying a truly low-risk cohort of patients who can be managed in primary care, and the approval of its use in primary care.^46^ The introduction, following recommendations in the 2022 Northern Ireland Cancer Strategy,^47^ of rapid diagnostic centres, and their perceived impact on GP referrals for cancer diagnoses (including EOCRC), also requires future study.^48^

A multifaceted approach is required to enable GPs to make more timely diagnoses and optimal use of secondary care services, and, ultimately, to improve the experiences and outcomes of patients with EOCRC.

## Data

The dataset relied on in this article is available from the corresponding author on request.
